# Characterization of *Listeria monocytogenes* Strains Isolated in Palermo (Sicily and Italy) during the Years 2018–2020 from Severe Cases of Listeriosis

**DOI:** 10.3390/antibiotics13010057

**Published:** 2024-01-06

**Authors:** Maria Rita Tricoli, Chiara Massaro, Ignazio Arrigo, Orazia Diquattro, Francesca Di Bernardo, Elena Galia, Mario Palermo, Teresa Fasciana, Anna Giammanco

**Affiliations:** 1 Department of Health Promotion, Mother and Child Care, Internal Medicine and Medical Specialities, University of Palermo, 90127 Palermo, Italyanna.giammanco@unipa.it (A.G.); 2 Laboratory of Microbiology, A. O. Ospedali Riuniti “Villa Sofia-Cervello”, 90100 Palermo, Italy; orazia.diquattro@villasofia.it; 3 Department of Microbiology and Virology, National Relevance and High Specialization Hospital Trust ARNAS Civico, Di Cristina, Benfratelli, 90127 Palermo, Italy; francesca.dibernardo@arnascivico.it; 4 Sicilian Health Department, Public Health and Environmental Risks Service, 90127 Palermo, Italy

**Keywords:** *Listeria monocytogenes*, serogroup, MLST, MVLST, antibiotic susceptibility

## Abstract

*Listeria monocytogenes* (LM), the etiological agent of listeriosis, can cause foodborne zoonosis. In this study, we characterized 23 strains that caused human severe listeriosis in Palermo (Sicily, Italy) during the period of 2018–2020. In addition, we assessed the phenotypic susceptibility of clinical isolates to antibiotics in accordance with EUCAST guidelines. The serogroup was determined through the use of PCR, while MLST and MVLST were identified through the sequencing of housekeeping genes. Finally, susceptibility to antibiotics was assessed by means of the Phoenix automatic system. Patients hospitalized with listeriosis were predominantly males (56% vs. 44% of females). The cases not associated with pregnancy included patients >65 years of age (60%), two of whom were affected by cancer, while cases associated with pregnancy included two pregnant women and three preterm infants. The data collected showed that the main pathologies shown by patients were meningitis (60.9%) and bacteremia (39.1%). The LM strains were isolated from the blood (52%), cerebrospinal fluid (26%), cerebrospinal fluid + blood (13%), blood + a nasal swab (4%), and ascitic fluid (4%). The predominant serogroup was IVb (96%), whereas only one strain belonged to serogroup IIa (4%). Among the strains with serotypes 4b, 4d, and 4e, ST2/VT21 (92%) and ST6/VT19 (4%) were determined, while only isolates with serotypes 1/2a and3a show ST155/VT45 (CC155). This study reveals the widespread circulation of a clinical strain (ST2/VT21) associated with suspected food contamination, demonstrating the importance of carrying out molecular epidemiological surveillance. Our clinical isolates were susceptible to the beta-lactams assayed, in agreement with the literature data.

## 1. Introduction

*Listeria monocytogenes* (LM), a Gram-positive bacillus widespread in the environment, is a pathogen responsible for human listeriosis, a serious food-borne zoonotic disease especially relevant to the elderly, immunosuppressed individuals, pregnant women and newborns [1,2,3,4]. Transmission generally occurs through the consumption of ready-to-eat (RTE) foods such as soft cheese, vegetables, fruit, meat, and salads [5,6,7].

The clinical manifestations of bacterial infections can take the form of non-invasive or invasive infections depending on the bacterial load, degree of virulence among strains, and the host’s immune system [8]. The non-invasive form usually occurs in immunocompetent subjects, causing typical symptoms of febrile gastroenteritis (with a high bacterial load of 10^9^ CFU/mL), while in immunosuppressed subjects such as the elderly, pregnant women, and newborns, LM may cause meningitis, meningoencephalitis, sepsis, and fetal infections [8]. Despite its relatively low incidence (1931 cases were reported in Europe in 2020), listeriosis is a serious health issue because it is associated with a high hospitalization (97.3%) and case fatality (12.8%) rate [9]. Furthermore, Italy, with 155 notified cases in 2020, has been classified by the ECDC as the fourth European country with the highest incidence rate of listeriosis [9]. Moreover, in 2020, the phenomenon was likely underestimated due latterly not only to the slowdown of laboratory investigations because of SARS-CoV-2 but also because the cases reported only concerned hospitalized patients; mandatory notification does not include non-invasive cases of listeriosis which resolve spontaneously.

Thus, listeriosis is subjected to constant epidemiological control through the collection of clinical strains and their phenotypic and molecular characterization [10].

Currently, 13 serotypes of LM have been identified, but only four (1/2a, 1/2b, 1/2c, and 4b) are responsible for most of the confirmed clinical cases in Europe and worldwide [10,11,12]. Serotype 4b appears to be the most virulent since it is responsible for most of the epidemics in humans, despite 1/2a being the most frequently isolated serotype from food [10,11,12,13].

Over the past few years, for typing, the techniques developed for improving the discriminatory power among LM strains are *Multilocus Sequence Typing* (MLST) and *Multi-Virulence-Locus Sequence Typing* (MvLST) based on the analyses of seven metabolic and six virulence housekeeping genes, respectively [14,15,16].

Unfortunately, in Sicily, listeriosis is underestimated because it is underreported; furthermore, the characterization of human clinical isolates is not determined.

This work aimed to evaluate molecular characteristics and phenotypic resistance to antibiotics predicted by the EUCAST 2023 guidelines of clinical strains from cases of listeriosis. We also considered the clinical and anamnestic information of patients in accordance with privacy policies. The strains, collected from serious cases of listeriosis coming from the main hospitals in Palermo from December 2018 to June 2020 at *the Department of Health Promotion, Mother and Child Care, Internal Medicine and Medical Specialties (PROMISE)* of the University of Palermo, Italy, were sent with a survey form to the Italian National Institute of Health (ISS) for collection. Local strains were characterized to determine serogroup, sequence typing (ST), and virulence type (VT; in addition, antimicrobial susceptibility testing was performed [13,14,15,16].

## 2. Materials and Methods

### 2.1. Data Collection of Patients

The 23 strains of LM species were isolated from biological samples of patients with invasive listeriosis, hospitalized in different hospitals in Palermo from December 2018 to June 2020, with sepsis and, in some cases, other complications as well.

Demographic and clinical characteristics of the listeriosis patients, risk factors (cancer, solid organ transplantation, inflammatory diseases, age >65 years, and pregnancy), date of collection of strains, and type of biological sample (blood culture, CSF, ascitic fluid, or nasal swab) were obtained from the ISS listeriosis notification questionnaire associated with each clinical strain. Data were collected anonymously in accordance with patient privacy. (Data available in Supplementary Table S1).

### 2.2. DNA Extraction of Clinical LM Strains

The clinical isolates, collected at ProMISE (Department of Health Promotion, Mother and Child Care, Internal Medicine and Medical Specialties), Policlinico “P. Giaccone” hospital in Palermo, were grown on a 5% sheep blood agar medium and identified and confirmed using matrix-assisted laser desorption/ionization–time of flight (MALDI-TOF) mass spectrometry (Bruker, Leipzig, Germany).

The genomic DNA of the LM isolates was extracted using the QIAprep 96 Plus Miniprep Kit (Qiagen, Hilden, Germany) following the manufacturer’s protocol. Furthermore, the DNA obtained was stored at −20 °C for subsequent analysis.

### 2.3. PCR Serogroup

All of the 23 LM strains were serogrouped by amplifying five target genes (prs, lmo0737, lmo1118, ORF2110, and ORF2819) using the primer and amplification conditions previously described [13]. This technique can discriminate 13 different serotypes previously grouped into 5 serogroups: IIa (1/2a and 3a), IIb (1/2b, 3b, and 7), IIc (1/2c, and 3c), IVb (4ab, 4b, 4d, and 4e), and finally IVa (4a and 4c) [13].

### 2.4. Multilocus Sequence Typing (MLST)

Multilocus sequence typing (MLST) was performed according to the protocol described by Moura et al., 2016. The PCR product of the single PCR of seven housekeeping genes (*abcZ*, *blgA*, *cat*, *dapE*, *dat*, *ldh*, and *lhkA*) was sequenced using a Genetic Analyzer (Applied Biosystems, Waltham, MA, USA) [17,18,19].

The sequence type (ST) and clonal complex (CC) of each LM clinical strain were assigned by inserting the sequences into the Institute Pasteur MLST database. Moreover, a phylogenetic tree was built using MEGAX software, V.7 [20], based on the concatenated DNA sequences (3288 bp) of the seven housekeeping genes.

The evolutionary history was deduced using the UPGMA method while the evolutionary distances were calculated using the maximum composite likelihood method [20].

### 2.5. Multi-Virulence-Locus Sequence Typing (MVLST)

Multi-virulence-locus sequence typing (MVLST) analyses were carried out using the primers and PCR protocols reported by Zhang et al. (2004) [15] to identify the virulence type (VT) and epidemic clones (EC) of the 23 LM strains. Six virulence genes (clpP, dal, inlB, inlC, lisR, and prfA) were amplified and then sequenced. The sequences obtained were concatenated following the order provided in the MvLST database [21] and then compared with multiple alignments to reference sequences. The phylogenetic tree was determined by analyzing the concatemers of each of the 23 LM strains through the use of the neighbor-joining method which includes the use of MegaX software [20].

### 2.6. Antibiotic Susceptibility of L. monocytogenes Isolates

Several antimicrobial agents exhibit in vitro activity against *L. monocytogenes* [22]. The primary therapy for invasive listeriosis consists of supportive therapy along with penicillin or ampicillin in combination with gentamicin. Trimethoprim/sulfamethoxazole can be used to treat patients who are allergic to penicillin or pregnant women [23].

The test was performed with the use of the Kirby–Bauer method (disc diffusion technique), and the antibiotics assayed, in accordance with European Committee on Antimicrobial Susceptibility Testing (EUCAST) guidelines, were: ampicillin (10 μg/disc), erythromycin (15 μg/disc), meropenem (10 μg/disc), penicillin G (10 μg/disc), and trimethoprim/sulfamethoxazole (25 µg/disc). Pure isolates were streaked tightly onto the surface of nutrient agar plates and incubated at 37 °C for 18 to 24 h. After incubation, the zones of inhibition surrounding each disc were observed. Interpretative breakpoints were determined always following the EUCAST guidelines [24,25].

## 3. Results

### 3.1. Demographic and Clinical Characteristics of Listeriosis Patients

LM strains were isolated from 23 hospitalized patients between 2018 and 2020 in Palermo, Italy, including thirteen males and ten females. The demographic and clinical data of our patients showed a greater number of cases in males than in females (13 vs. 10), showing the major susceptibility of man, as described in the literature [26].

**Figure 1:**
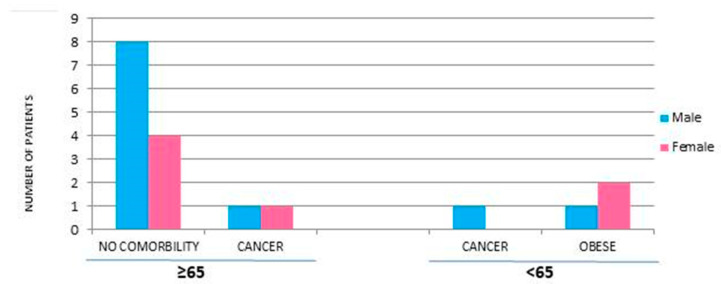
Risk factors and sex of patients with listeriosis not related to pregnancy.

Out of thirteen males, nine were over the age of 65, two were younger, and two were newborns who had acquired the infection at birth. Most of the males enrolled did not show other pathologies; in fact, only three had cancer (2/13) and one was affected by obesity (1/13) (Figure 1). However, 5/10 females were over the age of 65, 4/10 were under the age of 65, and there was one newborn girl. Among these females, one had cancer, two were obese, and three acquired the infection during pregnancy.

The three newborns showed meningitis, sepsis, or respiratory distress. All of the mothers were affected by intrauterine infection that caused premature birth, and two also showed meningitis and bacteremia.

**Figure 2:**
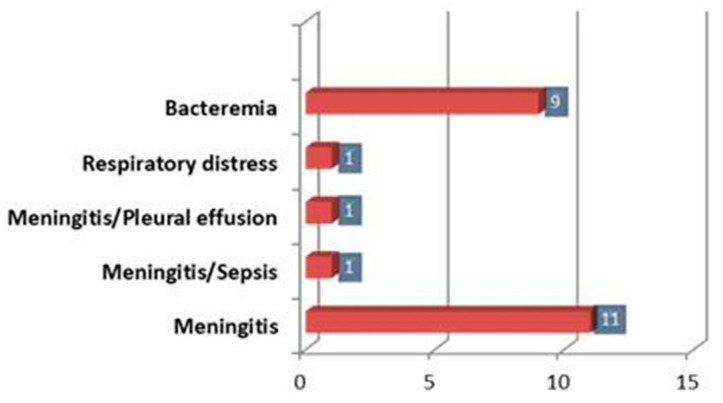
Clinical manifestation of the patients enrolled.

The most common clinical manifestation of listeriosis was meningitis, which was present in 14 patients (60.86%), two cases of which were associated with sepsis and pleural effusion. In comparison, 9/23 patients (39.13%) showed only bacteremia (Figure 2).

In addition, a total of four cases were associated with deaths, including patients undergoing immunosuppressive therapy (cancer and ulcerative colitis), a 49-year-old woman with meningitis, and a preterm newborn with meningitis.

**Figure 3:**
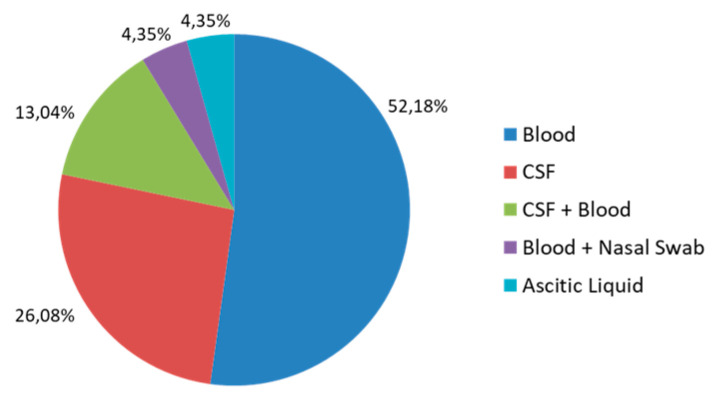
Source from which the LM isolates were obtained.

Moreover, twelve (52%) and six (26%) patients were positive in their blood and CSF cultures, respectively, while there were three cases (13%) positive in blood + CSF cultures. In addition, two other cases were associated with isolation from ascitic fluid (4%) and nasal swabs + blood (4%) (Figure 3).

### 3.2. Serogroup

The results showed the presence of only two different serogroups, IIa and IVb. Specifically, the prevalent serogroup was IVb associated with 95,6% of the isolates examined (*n* = 22), and only one strain belonged to IIa (4.4%).

While serogroup IVb clinical strains were isolated in newborns, pregnant women, the elderly, and cancer patients (subjects at risk of listeriosis) with meningitis, serogroup IIa clinical strains were isolated from one patient with ulcerative colitis with sepsis but without central nervous system involvement (data available in Supplementary Table S2).

### 3.3. Multilocus Sequence Typing (MLST)

The results obtained were according to those of the epidemiological studies published by Radoshevich et al. [27], in which CC 6, CC 2, and CC 155 represented the second, fourth, and eighth clonal complexes most represented among the clinical LM isolates, respectively.

Sequence type 2, shown in 21 strains (91%), was the most prevalent ST in the epidemic population circulating in Palermo in the period between December 2018 and July 2020.

Sequence type 6, considered hypervirulent ST in LM species, was present in one isolate (4%).

Type 155 with serogroup IIa was also identified in one case (4%).

### 3.4. Multi-Virulence-Locus Sequence Typing (MvLST)

Virulence type 2, isolated in 21 strains (91%), according to the MvLST database for LM species, has been associated with many epidemic outbreaks around the world, including those in the UK (1989), the USA (1979), France (2000), Germany (1953), New Zealand (1989), Denmark (1962), Canada (1950), and Italy (2012, contaminated food sources identified as ricotta-based products) [28].

Virulence type 19, shown in one case (4%), has been identified in Finland (1987, animals), Mexico (1999, the environment), France (1993 and 2003, pregnant women), and the USA (1996, 1997, 2002, 2004, and 2005 both from human, animal, and food clinical cases) [29].

Virulence type 45, also present in one strain (4%), has been isolated from human subjects in China (1991) and Canada (2002), while from food sources, it has been isolated in the USA (1999), New Zealand (1999), and China (2002) [30]. Moreover, VT 45 was prevalent in the LM strains isolated in Brazil during the period of 1979–2015 and is present in all food categories examined [31].

### 3.5. MLST and MvLST Analysis

Three different STs and VTs were obtained among all strains, which were included in three clonal complexes (CCs) and two epidemic clones (ECs).

Among the 3 STs, ST 2 (CC2)/VT 21 (ECIV) was predominant in 21 isolates (91%), followed by ST6(CC6)/VT19 (ECII) and ST155(CC155)/VT45, which were shown in one strain (4%) (Table 1). In particular, ST2 was predominantly isolated from blood samples (52%, 11/21) but also from CSF (14,28%, 3/21), ascetic liquid (4,76%, 1/21), and nasal swab (4,76%, 1/21) samples; only ST6 was isolated from CSF, while ST155 was isolated from blood.

The temporal distribution and frequency of STs from December 2018 to July 2020 showed a clear predominance of strains with ST2 probably originating from a single epidemic outbreak, while ST6 and ST155 appeared in only two isolated cases (Table 2) (data available in Supplementary Table S2).

**Figure 4:**
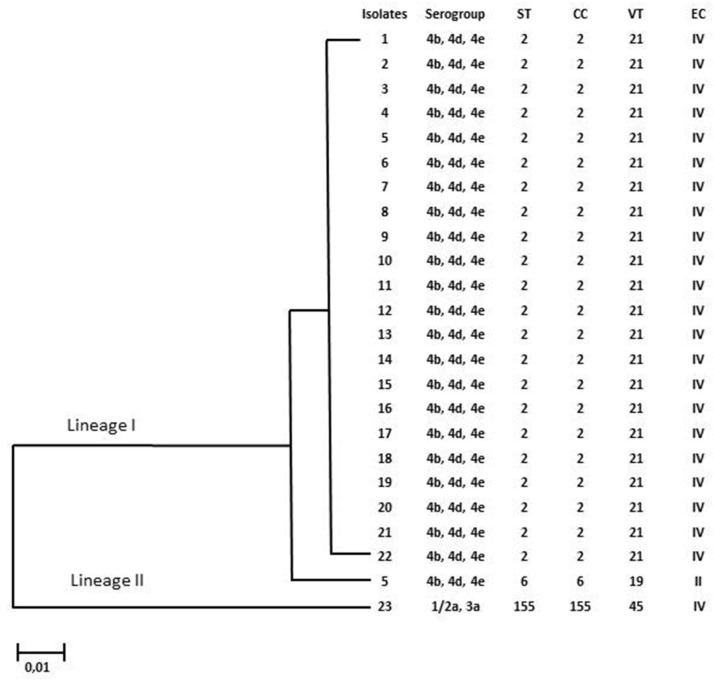
Characteristics of LM strains isolated from patients in Palermo, Italy. The neighbor-joining phylogeny tree as computed by MEGA.

All of the results concerning the molecular characterization of the clinical isolates are summarized in Figure 4, where a maximum likelihood-based phylogenetic tree was built based on the seven concatenated housekeeping gene sequences of 23 strains with Mega X software to analyze the phylogenetic relationship between the different STs.

### 3.6. Antibiotic Susceptibility of L. monocytogenes Isolates

All 23 isolates were found to be susceptible to ampicillin, erythromycin, and meropenem.

Ampicillin represents a valid empirical therapy in cases of meningitis, while definitive therapy with meropenem is associated with significantly higher 30-day mortality, as demonstrated by the literature [32].

However, oculoglandular listeriosis and listeria dermatitis generally responded to erythromycin.

Resistance to the other two antibiotics was as follows: penicillin G (*n* = 1; 4.3%) and trimethoprim/sulfamethoxazole (*n* = 10; 43.4%).

The resistance to only COT was not related to the different sequencing types; in fact, all strains belonging to type ST2 were found to be sensitive or resistant.

One case (sample 5) belonging to a different ST (ST6) was also resistant to penicillin.

However, this finding was not statistically significant due to the limited number of isolates with ST6.

No isolate was defined as multidrug-resistant (MDR). All of the results concerning the antibiotic susceptibility of the clinical isolates are summarized in Table 3.

## 4. Discussion

In this study, analyses of the characteristics of clinical LM isolates in Palermo during 2018–2020 was performed.

The results of this study highlight a higher number of cases of listeriosis in patients aged over 65, coinciding with the trend data published by the ECDC for the year 2020 [9].

Furthermore, serogroups 4b, 4d, and 4e were dominant in our study (95%), linked to cases of sepsis and/or meningitis, while only one strain belonging to serogroups 1/2a and 3a (4%) was associated with ulcerative proctocolitis without the involvement of the SNC, suggesting less virulence for the brain but more damage for the gastrointestinal tract.

Our results agree with ISS (the Italian National Institute of Health) laboratory surveillance reports [7] on listeriosis in Italy for the year 2020, which showed that the predominant serogroups were 4b, 4d, and 4e (43%) as compared to 1/2a and 3a (31%). They also agree with the epidemiological information of clinical strains circulating in the European population provided by the ECDC for 2019, where the strains with serogroups 4b, 4d, and 4e appear to be prevalent among human isolates compared to serogroup 1/2a and 3a [9].

According to Italian data, ST2 was the most frequent sequence type connected to Sicilian listeriosis. Since 1997, ST2 has been associated with the largest epidemic outbreak in Italy [33], with it being responsible for the hospitalization of 292 people (20% of patients) (children and primary school staff), caused by eating contaminated tuna and corn salad.

Numerous outbreaks originating from ST6 have been reported in the literature: in Switzerland in 2016 [34], in South Africa in 2017–2018 [35], and in five EU states (Austria, Denmark, Finland, Sweden, and the United Kingdom) in 2015–2018, probably linked to the consumption of frozen vegetables (probably corn), which caused 32 cases of human listeriosis and 6 deaths.

The most recent outbreak associated with ST6 of *L. monocytogenes* was recorded in Switzerland, with it being related to the consumption of contaminated cheese in the period of 2018–2020 which caused a total of 34 cases of listeriosis and 10 deaths [36].

With respect to sequence type 155, recent studies published in the literature show that it has a specific mutation in the *prfA* gene which determines the synthesis of a longer version of the master transcriptional activator of the virulence gene and consequently a lower expression of *inlA*, *inlB*, and *prfA* and no expression of *hly* and *actA*, causing a reduction in the virulence potential of these strains and an inability to cause meningitis [37]. In Italy, 22 isolates with ST155 were reported in Lombardy in the period from 2008 to 2014, with a peak of 10 cases in 2011 [38].

A close correlation has also been documented between ST and VT.

The prevalence of ST2/VT21 in the epidemic population from December 2018 to July 2020 in Palermo (Italy) indicated that many cases were likely related to epidemics that could not be identified without advanced molecular typing methods, which are crucial for identifying listeriosis outbreaks in which geographical and temporal spread is extensive.

Moreover, our study revealed the presence of a hypervirulent strain, ST6/VT19, associated with several epidemic outbreaks, particularly in Europe in 2015–2020, showing a wide and intercontinental diffusion [39]. Indeed, due to the worldwide commercialization of food products, hypervirulent strains spread rapidly over large geographic areas, causing multi-country outbreaks.

In accordance with the literature data [40,41], our results showed 43,4% of isolates as being resistant to trimethoprim/sulfamethoxazole (COT), which is considered appropriate to evaluate alternative empirical therapies in the case of penicillin allergies to increase the probability of patient survival. Only in one case, the COT resistance was associated with penicillin resistance.

The strains that showed the same ST were correlated with different antibiotic patterns and linked to clinical pictures with good relations. On the basis of our results, we could underline the necessity of ensuring public health and achieving food safety, predicting integrated health surveillance systems at the international level. These systems are considered essential in order to carry out large-scale epidemiological studies to rapidly identify and eliminate contaminated food products based on a comparison of clinical and food strain typing results.

## 5. Conclusions

In conclusion, molecular surveillance in hospital and school canteens is particularly important where there is a high number of subjects susceptible to listeriosis, including children, pregnant women, and immunosuppressed patients, in particular the elderly, or asymptomatic carriers of the microorganism [42].

In order to assess LM carrier status, a condition that could result in severe infection of the patient, it would be appropriate to screen pregnant women and the elderly through the use of vaginal swabs and fecal samples, respectively.

Our data support the possibility of a circulating clone associated with food contaminations in our geographic area.

The relevance of monitoring the spread of virulence genes between different LM strains was also demonstrated, not only to differentiate between low-virulence and hypervirulent ST but also to identify the source of contamination and new therapeutic approaches to contain their diffusion.

Obviously, additional studies have to be undertaken to evaluate the origin of the spread of clones, resistance to various chemical–physical stresses, and the degree of biofilm formation, which could facilitate their presence in the industry and help to contaminate food products and obviously the carrier status.

## Figures and Tables

**Table 1:** MLST, MvLST, and Serovar of the LM isolates.

STs	Clonal Complex (CC)	Lineage	Serovar	VTs	N° Isolates (%)
2	2	I	IVb, IVd, IVe	21	21 (92%)
6	6	II	IVb, IVd, IVe	19	1 (4%)
155	155	II	I/IIa, IIIa	45	1 (4%)

**Table 2:** Distribution of listeriosis cases from Winter 2018 to Summer 2020 in the region of Palermo (Italy).

	2018				2019				2020			
	WINTER	SPRING	SUMMER	AUTUMN	WINTER	SPRING	SUMMER	AUTUMN	WINTER	SPRING	SUMMER	AUTUMN
ST2	1	-	-	-	8	3	3	2	-	2	2	-
ST6	-	-	-	-	-	-	1	-	-	-	-	-
ST155	-	-	-	-	1	-	-	-	-	-	-	-

**Table 3:** Antibiotic susceptibility of L.M isolates through the use of the Kirby–Bauer method.

	AMPICILLIN	ERYTHROMYCIN	MEROPENEM	PENICILLIN G	TRIMETHOPRIM/ SULFAM
LM_01	S	S	S	S	S
LM_02	S	S	S	S	S
LM_03	S	S	S	S	R
LM_04	S	S	S	S	R
LM_05	S	S	S	R	R
LM_06	S	S	S	S	I
LM_07	S	S	S	S	R
LM_08	S	S	S	S	S
LM_09	S	S	S	S	R
LM_10	S	S	S	S	S
LM_11	S	S	S	S	S
LM_12	S	S	S	S	R
LM_13	S	S	S	S	R
LM_14	S	S	S	S	R
LM_15	S	S	S	S	S
LM_16	S	S	S	S	S
LM_17	S	S	S	S	S
LM_18	S	S	S	S	R
LM_19	S	S	S	S	R
LM_20	S	S	S	I	S
LM_21	S	S	S	S	S
LM_22	S	S	S	S	S
LM_23	S	S	S	S	S

## Data Availability

All data used in the study were anonymized, according to the requirements set by the Italian Data Protection Code (leg. Decree 196/2003), and the general authorizations were issued by the Data Protection Authority. Approval by the Ethics Committee was obtained by Azienda Ospedaliera Universitaria Policlinico “P. Giaccone” of Palermo (protocols n°07/2019).

## Supplementary Materials

The following supporting information can be downloaded at: https://www.mdpi.com/article/10.3390/antibiotics13010057/s1. Table S1: Information on the 23 listeriosis patients; Table S2: MLST, MvLST, and serovar of 23 LM isolates.

## References

[1] Swaminathan B., Gerner-Smidt P. (2007). The epidemiology of human listeriosis. Microbes Infect..

[2] Lake F.B., van Overbeek L.S., Baars J.J., Koomen J., Abee T., Besten H.M.D. (2021). Genomic characteristics of Listeria monocytogenes isolated during mushroom (*Agaricus bisporus*) production and processing. Int. J. Food Microbiol..

[3] Thomas M.K., Murray R., Flockhart L., Pintar K., Pollari F., Fazil A., Nesbitt A., Marshall B., Racicot M., Comeau G. (2013). Estimates of the Burden of Foodborne Illness in Canada for 30 Specified Pathogens and Unspecified Agents, Circa 2006. Foodborne Pathog. Dis..

[4] Zhang X., Liu Y., Zhang P., Niu Y., Chen Q., Ma X. (2021). Genomic Characterization of Clinical Listeria monocytogenes Isolates in Beijing, China. Front. Microbiol..

[5] Cartwright E.J., Jackson K.A., Johnson S.D., Graves L.M., Silk B.J., Mahon B.E. (2013). Listeriosis Outbreaks and Associated Food Vehicles, United States, 1998–2008. Emerg. Infect. Dis..

[6] Moravkova M., Verbikova V., Michna V., Babak V., Cahlikova H., Karpiskova R., Kralik P. (2017). Detection and Quantification of Listeria monocytogenes in Ready-to-eat Vegetables, Frozen Vegetables and Sprouts Examined by Culture Methods and Real-time PCR. J. Food Nutr. Res..

[7] https://www.epicentro.iss.it/listeria/.

[8] Schlech W.F., Acheson D. (2000). Foodborne Listeriosis. Clin. Infect. Dis..

[9] https://www.ecdc.europa.eu/en/surveillance-atlas-infectious-diseases.

[10] Hedberg C. (2006). Listeria in Europe: The need for a European surveillance network is growing. Eurosurveillance.

[11] McLauchlin J., Mitchell R., Smerdon W.J., Jewell K. (2003). Listeria monocytogenes, and listeriosis: A review of hazard characterization for use in microbiological risk assessment of foods. Int. J. Food Microbiol..

[12] Orsi R.H., den Bakker H.C., Wiedmann M. (2011). Listeria monocytogenes lineages: Genomics, evolution, ecology and phenotypic characteristics. Int. J. Med. Microbiol..

[13] Doumith M., Buchrieser C., Glaser P., Jacquet C., Martin P. (2004). Differentiation of the Major Listeria monocytogenes Serovars by Multiplex PCR. J. Clin. Microbiol..

[14] Salcedo C., Arreaza L., Alcalá B., de la Fuente L., Vázquez J.A., Kazor C.E., Mitchell P.M., Lee A.M., Stokes L.N., Loesche W.J. (2003). Development of a Multilocus Sequence Typing Method for Analysis of *Listeria monocytogenes* Clones. J. Clin. Microbiol..

[15] Zhang W., Jayarao B.M., Knabel S.J. (2004). Multi-Virulence-Locus Sequence Typing of *Listeria monocytogenes*. Appl. Environ. Microbiol..

[16] Zhang Y., Dong S., Chen H., Chen J., Zhang J., Zhang Z., Yang Y., Xu Z., Zhan L., Mei L. (2019). Prevalence, Genotypic Characteristics and Antibiotic Resistance of Listeria monocytogenes From Retail Foods in Bulk in Zhejiang Province, China. Front. Microbiol..

[17] Wu S., Wu Q., Zhang J., Chen M., Guo W. (2016). Analysis of Multilocus Sequence Typing and Virulence Characterization of Listeria monocytogenes Isolates from Chinese Retail Ready-to-Eat Food. Front. Microbiol..

[18] Moura A., Criscuolo A., Pouseele H., Maury M.M., Leclercq A., Tarr C. (2016). Whole genome-based population biology and epidemiological surveillance of *Listeria monocytogenes*. Nat. Microbiol..

[19] https://bigsdb.pasteur.fr/listeria/listeria.html.

[20] https://www.megasoftware.net.

[21] https://sites.google.com/site/mvlstdatabase/home.

[22] Marco F., Almela M., Nolla-Salas J., Coll P., Gasser I., Ferrer M.D., de Simon M. (2000). In vitro activities of 22 antimicrobial agents against Listeria monocytogenes strains isolated in Barcelona, Spain. Diagn. Microbiol. Infect. Dis..

[23] https://www.cdc.gov/listeria/risk-groups/pregnant-women.html.

[24] Bauer A.W., Kirby W.M.M., Sherris J.C., Turck M. (1966). Antibiotic Susceptibility Testing by a Standardized Single Disk Method. Am. J. Clin. Pathol..

[25] https://www.eucast.org/.

[26] Huang Y.-T., Kuo Y.-W., Lee M.-R., Tsai Y.-H., Teng L.-J., Tsai M.-S., Liao C.-H., Hsueh P.-R. (2021). Clinical and molecular epidemiology of human listeriosis in Taiwan. Int. J. Infect. Dis..

[27] Radoshevich L., Cossart P. (2018). Listeria monocytogenes: Towards a complete picture of its physiology and pathogenesis. Nat. Rev. Microbiol..

[28] https://www.sites.google.com/site/mvlstdatabase/isolates-information-sorted-by-vts/isolates-belonging-to-vt21.

[29] https://www.sites.google.com/site/mvlstdatabase/isolates-information-sorted-by-vts/isolates-belonging-to-vt19.

[30] https://www.sites.google.com/site/mvlstdatabase/isolates-information-sorted-by-vts/isolates-belonging-to-vt45.

[31] da Silva D.A.F., Vallim D.C., Rosas C.D.O., de Mello V.M., Brandão M.L.L., de Filippis I. (2020). Genetic diversity of Listeria monocytogenes serotype 1/2a strains collected in Brazil by Multi-Virulence-Locus Sequence Typing. Lett. Appl. Microbiol..

[32] Thønnings S., Knudsen J.D., Schønheyder H.C., Søgaard M., Arpi M., Gradel K.O., Østergaard C., Jensen U., Koch K., Pinholt M. (2016). Antibiotic treatment and mortality in patients with Listeria monocytogenes meningitis or bacteraemia. Clin. Microbiol. Infect..

[33] Aureli P., Fiorucci G.C., Caroli D., Marchiaro G., Novara O., Leone L., Salmaso S. (2000). An Outbreak of Febrile Gastroenteritis Associated with Corn Contaminated by *Listeria monocytogenes*. N. Engl. J. Med..

[34] Althaus D., Jermini M., Giannini P., Martinetti G., Reinholz D., Nüesch-Inderbinen M., Lehner A., Stephan R. (2017). Local outbreak of Listeria monocytogenes serotype 4b sequence type 6 due to contaminated meat pâté. Foodborne Pathog. Dis..

[35] Smith A.M., Tau N.P., Smouse S.L., Allam M., Ismail A., Ramalwa N.R., Disenyeng B., Ngomane M., Thomas J. (2019). Outbreak of Listeria monocytogenes in South Africa, 2017–2018: Laboratory activities and experiences associated with whole-genome sequencing analysis of isolates. Foodborne Pathog. Dis..

[36] Nüesch-Inderbinen M., Bloemberg G.V., Müller A., Stevens M.J., Cernela N., Kollöffel B., Stephan R. (2021). Listeriosis Caused by Persistence of *Listeria monocytogenes* Serotype 4b Sequence Type 6 in Cheese Production Environment. Emerg. Infect. Dis..

[37] Wagner E., Zaiser A., Leitner R., Quijada N.M., Pracser N., Pietzka A., Ruppitsch W., Schmitz-Esser S., Wagner M., Rychli K. (2020). Virulence characterization and comparative genomics of Listeria monocytogenes sequence type 155 strains. BMC Genom..

[38] Amato E. (2016). Epidemiologia Della Listeriosi Invasiva in Regione Lombardia: Identificazione dei Genotipi Emergent.

[39] Guidi F., Centorotola G., Chiaverini A., Iannetti L., Schirone M., Visciano P., Cornacchia A., Scattolini S., Pomilio F., D’Alterio N. (2023). The Slaughterhouse as Hotspot of CC1 and CC6 *Listeria monocytogenes* Strains with Hypervirulent Profiles in an Integrated Poultry Chain of Italy. Microorganisms.

[40] Korsak D., Krawczyk-Balska A., Hutchinson H., Muñoz-Vargas L., Feicht S., Habing G., Colonna W., Brehm-Stecher B., Shetty K., Iii A.P. (2017). Identification of the Molecular Mechanism of Trimethoprim Resistance in *Listeria monocytogenes*. Foodborne Pathog. Dis..

[41] Srinivasan V., Nam H.M., Nguyen L.T., Tamilselvam B., Murinda S.E., Oliver S.P. (2005). Prevalence of Antimicrobial Resistance Genes in *Listeria monocytogenes* Isolated from Dairy Farms. Foodborne Pathog. Dis..

[42] Davis M.L., Ricke S.C., Donaldson J.R. (2019). Establishment of *Listeria monocytogenes* in the Gastrointestinal Tract. Microorganisms.

