# Supplementary material for: Characterization of Listeria monocytogenes Strains Isolated in Palermo (Sicily and Italy) during the Years 2018–2020 from Severe Cases of Listeriosis
Source: Antibiotics (Basel). 2024 Jan 6;13(1):57. doi: 10.3390/antibiotics13010057 (PMC10812810; doi:10.3390/antibiotics13010057)
Supplement: Supplementary file 1 [file antibiotics-13-00057-s001.zip › antibiotics-2727130-supplementary.pdf]

SUPPLEMENTARY INFORMATION

TABLE S1. Information of the 23 Listeriosis patients.

Patient n°	Sex	Age	Culture Sites	Risk Factors	Main Diagnose
1	F	82y	CSF	>65	Meningitis, pleural effusion
2	F	65y	Blood	≥65<br>Cancer	Bacteremia, Cough, abdominal pain
3	F	49y	Blood + CSF	Obese	Meningitis
4	F	83y	CSF	>65	Meningitis
5	M	81y	CSF	>65	Meningitis
6	F	55y	Blood	Obese	Meningitis
7	F	28y	Blood + CSF	Pregnancy-associated	Meningitis<br>Mother: Vomiting, Fever, Birthinduction
8	M	80y	Ascitic fluid	>65	Meningitis
9	M	1d	Blood.	Pregnancy-associated	Meningitis<br>Mother: Sepsis, meningitis
10	M	72y	CSF	>65	Meningitis
11	F	1d	Blood.	Pregnancy-associated	Bacteremia and meningitis<br>Mother: track alteration and severeasphyxia
12	F	78y	CSF	> 65	Meningitis
13	M	73y	Blood	> 65	Meningitis, Fever
14	M	68y	Blood	> 65	Bacteremia
15	M	1d	Nasal swab	Pregnancy-associated	Respiratory distress
16	M	66y	Blood + CSF	> 65<br>Leukemia	Bacteremia
17	M	37y	CSF	Obese	Meningitis
18	F	73y	Blood	>65	Bacteremia
19	M	78y	Blood	>65	Bacteremia

20	M	51y	Blood	Lung Cancer	Bacteremia, fever and cough
21	F	35y	Blood	Pregnancy-associated	Bacteremia
22	M	71y	Blood.	>65	Bacteremia
23	M	73y	Blood.	>65	Bacteremia, Rectorrhagia from ulcerative colitis

TABLE S2. MLST, MvLST and serovar of 23 LM isolates.

		MLST									MvLST							
**SAMPLE**	**Serovar**	**abc-z**	**bgla**	**cat**	**dap-e**	**dat**	**Ldh1**	**lhka**	**ST**	**CC**	**clpP**	**dal**	**inlB**	**inlC**	**lisR**	**prfA**	**VT**	**EC**
**1**	IVb	1	1	11	11	2	1	5	2	2	4	5	14	14	3	2	21	IV
**2**	IVb	1	1	11	11	2	1	5	2	2	4	5	14	14	3	2	21	IV
**3**	IVb	1	1	11	11	2	1	5	2	2	4	5	14	14	3	2	21	IV
**4**	IVb	1	1	11	11	2	1	5	2	2	4	5	14	14	3	2	21	IV
**5**	IVb	3	9	9	3	3	1	5	6	6	4	10	13	10	2	2	19	II
**6**	IVb	1	1	11	3	3	1	5	2	2	4	5	14	14	3	2	21	IV
**7**	IVb	1	1	11	11	2	1	5	2	2	4	5	14	14	3	2	21	IV
**8**	IVb	1	1	11	11	2	1	5	2	2	4	5	14	14	3	2	21	IV
**9**	IVb	1	1	11	11	2	1	5	2	2	4	5	14	14	3	2	21	IV
**10**	IVb	1	1	11	11	2	1	5	2	2	4	5	14	14	3	2	21	IV
**11**	IVb	1	11	11	11	2	1	5	2	2	4	5	14	14	3	2	21	IV
**12**	IVb	1	1	11	11	2	1	5	2	2	4	5	14	14	3	2	21	IV
**13**	IVb	1	1	11	11	2	1	5	2	2	4	5	14	14	3	2	21	IV
**14**	IVb	1	1	11	11	2	1	5	2	2	4	5	14	14	3	2	21	IV
**15**	IVb	1	1	11	11	2	1	5	2	2	4	5	14	14	3	2	21	IV
**16**	IVb	1	1	11	11	2	1	5	2	2	4	5	14	14	3	2	21	IV
**17**	IVb	1	1	11	11	2	1	5	2	2	4	5	14	14	3	2	21	IV
**18**	IVb	1	1	11	11	2	1	5	2	2	4	5	14	14	3	2	21	IV
**19**	IVb	1	1	11	11	2	1	5	2	2	4	5	14	14	3	2	21	IV
**20**	IVb	1	1	11	11	2	1	5	2	2	4	5	14	14	3	2	21	IV
**21**	IVb	1	1	11	11	2	1	5	2	2	4	5	14	14	3	2	21	IV
**22**	IVb	1	1	11	11	2	1	5	2	2	4	5	14	14	3	2	21	IV
**23**	IIa	7	10	16	7	5	2	1	155	155	31	19	5	9	12	1	45	IV
